# Effects of Prior Metformin Use on Stroke Outcomes in Diabetes Patients with Acute Ischemic Stroke Receiving Endovascular Treatment

**DOI:** 10.3390/biomedicines12040745

**Published:** 2024-03-27

**Authors:** Chulho Kim, Yejin Kim, Jong-Hee Sohn, Joo Hye Sung, Sang-Won Han, Minwoo Lee, Yerim Kim, Jae Jun Lee, Hee Jung Mo, Kyung-Ho Yu, Sang-Hwa Lee

**Affiliations:** 1 Department of Neurology, Chuncheon Sacred Heart Hospital, Hallym University College of Medicine, Chuncheon 24252, Republic of Korea; gumdol52@naver.com (C.K.); deepfoci@hallym.or.kr (J.-H.S.); centertruth@naver.com (J.H.S.); rabiting0@daum.net (S.-W.H.); 2 Institute of New Frontier Research Team, Hallym University, Chuncheon 24252, Republic of Korea; hiruizhen@naver.com (Y.K.); iloveu59@hallym.or.kr (J.J.L.); 3 Department of Neurology, Hallym Sacred Heart Hospital, Hallym University College of Medicine, Anyang 14068, Republic of Korea; minwoo.lee.md@gmail.com (M.L.); ykh1030@hallym.or.kr (K.-H.Y.); 4 Department of Neurology, Kangdong Sacred Heart Hospital, Hallym University College of Medicine, Seoul 05355, Republic of Korea; brainyrk@gmail.com; 5 Department of Anesthesiology and Pain Medicine, Chuncheon Sacred Heart Hospital, Hallym University College of Medicine, Chuncheon 24252, Republic of Korea; 6 Department of Neurology, Dongtan Sacred Heart Hospital, Hallym University College of Medicine, Hwaseong 18450, Republic of Korea; hjungmo@gmail.com

**Keywords:** DM, prior metformin use, endovascular treatment, hemorrhagic transformation, stroke progression

## Abstract

Diabetes mellitus (DM) predisposes individuals to vascular injury, leading to poor outcomes after ischemic stroke and symptomatic hemorrhagic transformation (SHT) after thrombolytic and endovascular treatment (EVT). Metformin (MET), an oral antidiabetic drug, has shown potential neuroprotective effects, but its impact on stroke prognosis in DM patients undergoing EVT remains unclear. In a multicenter study, 231 patients with DM undergoing EVT for acute ischemic stroke were enrolled. Prior MET use was identified, and patients were stratified into MET+ and MET− groups. Demographics, clinical data, and outcomes were compared between groups. Multivariate analysis was used to assess the effect of MET on stroke prognosis. Of the enrolled patients, 59.3% were previously on MET. MET+ patients had lower initial infarct volumes and NIHSS scores compared to MET-taking patients. Multivariate analysis showed that MET+ was associated with a lower risk of stroke progression and SHT (with stroke progression as follows: odd ratio [OR] 0.24, 95% confidence interval [CI] [0.12–0.48], *p* < 0.001; SHT: OR 0.33, 95% CI [0.14–0.75], *p* = 0.01) and was also associated with better 3-month functional outcomes (mRS 0–2) after EVT. Prestroke MET use in DM patients undergoing EVT is associated with improved stroke prognosis, including reduced risk of stroke progression and SHT and better functional outcomes. These findings suggest the potential neuroprotective role of MET in this population and highlight its clinical utility as an adjunctive therapy in the management of ischemic stroke. Further research is warranted to elucidate the underlying mechanisms and to optimize MET therapy in this setting.

## 1. Introduction

Diabetes mellitus (DM) is associated with an increased risk of vascular disease, particularly ischemic stroke, which poses significant challenges for clinical management and patient prognosis. Ischemic strokes in diabetic patients are often more severe and have worse recovery outcomes than those in non-diabetic stroke patients due to the multiple vascular complications caused by hyperglycemia. The pathological hallmark of diabetes mellitus (DM) involves vasculature, resulting in both microvascular and macrovascular injury [1]. Therefore, DM is associated with an unfavorable functional outcome after ischemic stroke and with more symptomatic hemorrhagic transformation (SHT) after intravenous thrombolysis (IVT) and endovascular treatment (EVT) [2,3,4]. Because previous studies have reported poor stroke prognosis after EVT due to multiple mechanisms (reperfusion injury, reocclusion, hypoperfusion, etc.), there is a need for therapeutic strategies to improve prognosis after EVT in patients with DM [5,6].

Metformin (MET) is an oral antidiabetic drug widely used as a first-line treatment for type 2 DM [7,8]. MET has gained attention as a treatment strategy for diabetes due to its hypoglycemic effects and potential neuroprotective properties. It is suggested that MET inhibits the inflammatory response, stimulates vascular endothelial growth and angiogenesis, and then attenuates blood–brain barrier (BBB) injury [9,10]. Experimental studies suggest that the neuroprotective effects of MET treatment are mediated through the modification of 5-adenosine monophosphate-activated protein kinase (AMPK) activity [11,12]. In addition, studies have shown that MET can improve angiogenesis and BBB integrity. These mechanisms have the potential to reduce ischemic injury and facilitate recovery after stroke. Therefore, the beneficial effects of MET on ischemic and hemorrhagic stroke have recently been reported in clinical trials [9,13,14,15,16]. These studies have shown that stroke patients with diabetes who take MET have a favorable outcome, and a recent study showed a positive effect of MET use on stroke outcomes in patients with ischemic stroke who underwent IVT. However, the prognostic impact of prestroke MET use in DM patients receiving EVT has not been described.

However, the variable outcomes of diabetic patients undergoing EVT highlight the need for additional treatments that may improve the benefits of revascularization. Because large vessel occlusion is associated with severe neurological deterioration, investigating the impact of prestroke MET use on the prognosis of patients with ischemic stroke undergoing EVT may have important clinical implications. With the increasing prevalence of diabetes and the increasing use of EVT in stroke management, it is important to investigate the potential benefits of MET beyond glycemic control.

This study aims to address this gap in the literature by evaluating the impact of prior MET use on stroke outcomes in DM patients undergoing EVT. By utilizing a multicenter cohort, we evaluate whether the use of MET is linked to an improved prognosis following ischemic stroke.

## 2. Methods

### 2.1. Study Design

This study used a multicenter, observational cohort design to evaluate the impact of prior MET use on stroke outcomes in patients with DM undergoing EVT for acute ischemic stroke. The study was conducted at four university-affiliated hospitals using a web-based registry database to identify eligible patients. Our approach was designed to examine real-world outcomes in a diverse patient population, reflecting the variability seen in clinical practice. The observational nature of the study allowed us to assess the association between prestroke MET use and stroke outcomes without the ethical and practical challenges of randomizing treatments in the context of an acute medical emergency. The comparison of outcomes between patients with and without prior MET use aimed to infer the potential neuroprotective effects of MET in a real-world setting.

### 2.2. Study Population

We consecutively enrolled patients with acute ischemic stroke from March 2015 to September 2023 in four university-affiliated multicenter web-based registry databases. For the purpose of this study, we identified patients with acute ischemic stroke with large artery occlusion of the anterior circulation who received EVT. Among these EVT-treated patients, we identified patients who were diagnosed with type 2 DM before stroke or who had an admission of HbA1c ≥ 6.5% at the time of stroke. We excluded the following: (1) patients with a modified Rankin Scale (mRS) score ≥ 2 before stroke, (2) patients without initial brain computed tomography (CT) or magnetic resonance imaging (MRI) scan within 24 h of stroke onset, (3) patients with an Alberta Stroke Program Early CT score > 6, and (4) patients without a 3-month mRS score.

### 2.3. Data Collection and Definition of Parameters

We obtained demographic, clinical, laboratory, and outcome data directly from the web-based registry databases of the four institutions. Previous MET use was defined as the use of any antidiabetic drug of the biguanide class before hospitalization and was identified by neurologists and the nationwide Drug Utilizing Review system [17]. Seventeen patients were divided into two groups, including a group of patients who used MET before stroke (MET+) and a group of patients who did not use MET (MET−). The MET+ group was defined as undergoing previous MET monotherapy and MET in combination with other antidiabetic agents. The MET− group was defined as receiving other antidiabetic agents and no antidiabetic agents. Collateral status was categorized as good, fair, or poor according to the Calgary Stroke Program imaging protocols using multiphasic CT angiography [18]. Collateral status was quantified by two experienced vascular neurologists (C Kim and S-H Lee) in a double-blinded manner (interclass correlation coefficient, 0.90; *p* < 0.001). Infarct volume based on diffusion-weighted imaging was calculated using Medical Image Processing and Visualization software (version 7.3.0, National Institutes of Health, Bethesda, MD, USA). In addition, to evaluate the effect of MET dose on stroke outcomes, we categorized the MET dose as low (<1000 mg daily), moderate (1000–2000 mg daily), and high (>2000 mg daily).

The primary outcome measure was early neurological deterioration (END). In addition, END was defined as an increase of at least 1 point in motor performance or a National Institute of Health Stroke Scale (NIHSS) total score deterioration of ≥2 points within 3 days of hospitalization compared to the initial NIHSS score. In this study, we categorized the etiology of END as stroke progression (END-prog) and symptomatic hemorrhagic transformation (END-SHT) after EVT [19]. Hemorrhagic transformation was defined according to the European Cooperative Acute Stroke Study criteria [20]. Two vascular neurologists (C Kim and S-H Lee) reviewed the END data to confirm END-prog and END-SHT in a double-blind manner (ICC, 0.89; *p* > 0.001). The secondary outcome measure was the functional outcome defined as a 3-month mRS score from 0 to 2 after EVT.

### 2.4. Statistical Analysis

We hypothesized that prior MET use may decrease the risk of END after EVT. Descriptive statistics include the mean and standard deviation (SD) for continuous variables, the median and interquartile range for ordinal variables, and the number and percentage of the total for categorical variables. These were comparisons between the MET+ and MET− groups using Pearson’s chi-squared test for categorical variables and Student’s *t*-test or Mann–Whitney U test for continuous variables.

To assess the independent effects of prior MET use on outcome measures, we performed binary logistic regression analysis; variables were selected for adjustment in the multivariable analysis if their *p* values were <0.2 in comparison to prior MET use and if their associations with each outcome variable were clinically plausible. Crude and adjusted odds ratios (ORs) and 95% confidence intervals (CIs) were calculated.

As a sensitivity analysis, we performed multivariate analysis to determine whether stroke outcomes could vary by MET dose. We evaluated the effect of the categorized MET dose on primary and secondary outcomes using binary logistic regression analysis. In addition, we also evaluated the effect of MET monotherapy (excluding MET multitherapy) on the outcomes.

## 3. Results

**Figure 1:**
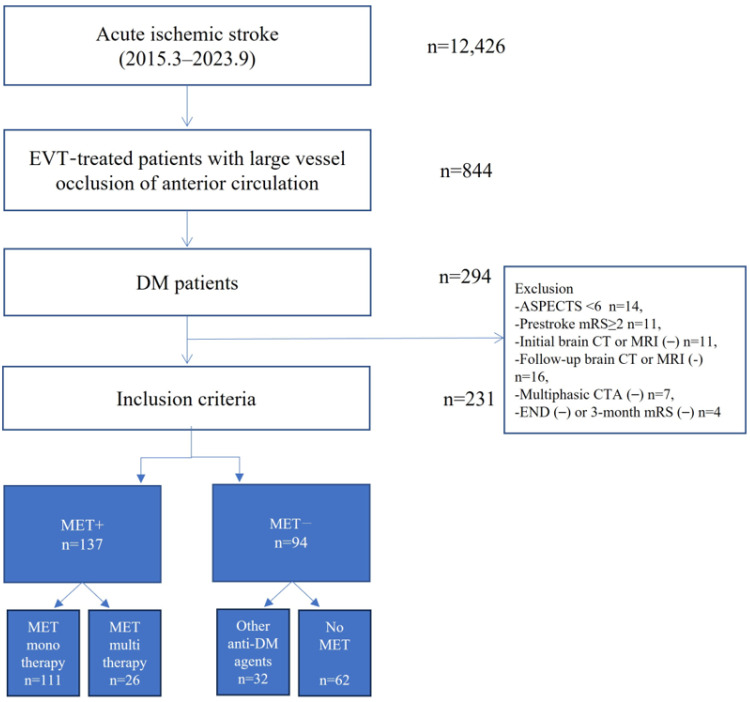
Flow chart of this study.

**Figure 2:**
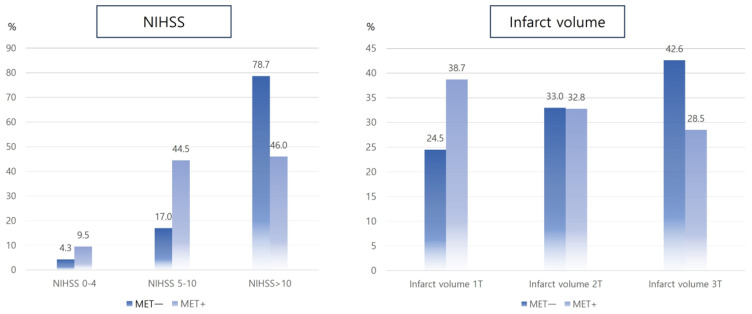
Comparison of initial NIHSS and infarct volume between MET+ and MET− groups. Abbreviations: NIHSS, National Institute of Health Stroke Scale; MET+, prestroke metformin use; 1T, first tertiles; 2T, second tertiles; 3T, third tertiles.

Of the 12,426 consecutive patients with acute ischemic stroke, 844 (6.8%) received EVT for large vessel occlusion in the anterior circulation. Of the 844 patients, 231 who met the inclusion criteria were enrolled in the study (Figure 1). Of the enrolled patients, 59.3% (137/231) had prior MET use. Of those with prior MET use, 81.0% (111/137) were undertaking MET monotherapy. MET multitherapy involved MET plus other antidiabetic agents such as glimepiride (*n* = 7), dipeptidyl peptidase-4 inhibitor (*n* = 14), and sodium-glucose cotransporter inhibitor (*n* = 5). In general, demographic, clinical and laboratory variables did not differ between the MET+ and MET− groups (Table 1). The MET+ group had a lower initial infarct volume than the MET− group (13.0 mL vs. 30.5 mL *p* = 0.04). In addition, the MET+ group had a trend toward lower initial NIHSS and tertiles of initial infarct volume than the MET− group (Figure 2). Laboratory variables, including glycated hemoglobin (HbA1c), initial random glucose, creatinine, and low-density lipoprotein cholesterol levels, were not different between the MET+ and MET− groups. According to the MET dose, the HbA1c level had an increasing trend with a higher MET dose (Supplementary Figure S1).

In multivariate analysis, MET+ decreased the risk of END-prog and END-SHT (END-prog as follows: OR 0.24, 95% CI [0.12–0.48], *p* < 0.001; END-SHT: OR 0.33, 95% CI [0.14–0.75], *p* = 0.01). For the secondary outcome measure, MET+ was associated with a 3-month mRS score 0–2 (OR 2.80, 95% CI [1.27–6.21], *p* = 0.01, Table 2).

In sensitivity analysis, the MET dose did not show a dose-dependent effect on END-prog, END-SHT or the 3-month mRS score 0–2 (Table 3, Supplementary Table S1). When only patients receiving MET monotherapy were analyzed, the effect of MET monotherapy on the primary and secondary endpoints remained (END-prog: OR 0.20, 95% CI [0.09–0.43]; END-SHT: OR 0.25, 95% CI [0.10–0.64]; 3-month mRS score 0–2: OR 2.92, 95% CI [1.25–6.86], Supplementary Table S2).

## 4. Discussion

The main findings of this study were as follows: (1) subjects with prior MET use before EVT had lower initial NIHSS and infarct volume than those without prior MET use, (2) prior MET use could reduce the risk of END-prog and END-SHT after EVT, and (3) prior MET use was associated with a 3-month mRS of 0 to 2 after EVT in patients with type 2 DM.

Our study highlights the importance of considering MET use before stroke as a potential predictor of stroke prognosis in patients undergoing EVT. A Japanese study (*n* = 355) showed that MET pretreatment was associated with mild stroke but not with functional outcome (mRS 0 to 2) [9]. Consistent with our findings, a study with a large population (*n* = 1919) receiving IVT and the overall ischemic stroke population (*n* = 7587) reported the beneficial effects of MET in ischemic stroke [14,16]. In large cardiovascular disease cohort studies, MET use is associated with lower rates of composite outcomes and death in type 2 DM [21,22]. Our study specifically focuses on its effects in the context of EVT, filling an important gap in the literature. The observed association between prestroke MET use and improved functional outcomes, as measured by the mRS at 3 months, further supports the potential clinical utility of MET in this setting.

More importantly, we observed that early stroke prognosis (END) and delayed functional prognosis were more favorable in MET+ than MET-patients after EVT. Given the lower initial NIHSS and infarct volume in MET+ patients, MET exerts a protective effect even before stroke onset and EVT. Therefore, we could assume that the effect of MET on delayed functional outcomes at 3 months is caused by lower stroke severity, infarct volume, and the rate of END after EVT, as in our study. In multivariate analysis, glycemic control (HbA1c) and initial glucose level were not associated with stroke outcomes in our study. We cautiously suggested that this result also supports our main findings that prior MET use alone could affect stroke outcomes after EVT. In addition, the rate of MET multitherapy in this study was relatively lower (19%) than in other studies (40%) [13]. A previous study showed that MET multitherapy had a greater benefit for better prognosis after acute ischemic stroke [13]. Although we could not evaluate the effect of MET multitherapy on stroke outcomes after EVT due to a small sample size, our sensitivity analysis uncovered at least part of the effect of MET monotherapy on early and delayed prognosis after the EVT is made robust. Further studies are needed to evaluate the effect of MET stratified by dose and in combination with other antidiabetic agents on stroke outcomes after EVT.

Our study adds to the growing body of evidence supporting the neuroprotective properties of MET in ischemic stroke. One possible mechanism of MET-induced neuroprotective effects in the context of ischemic stroke has been linked to its chronic activation of AMPK as a central regulator of cellular energy homeostasis and metabolism [23]. AMPK is a novel regulator of angiogenesis via endothelial cell migration and differentiation under hypoxic conditions [24,25]. In addition, activated AMPK may promote endothelial function by phosphorylating endothelial nitric oxide synthase [26]. Because AMPK activation is mediated by MET, MET may reduce oxidative stress, enhance angiogenesis, and attenuate BBB disruption [10,27]. In addition, activated AMPK via chronic MET uses preconditioned brain tissue with higher lactate levels, making the brain less vulnerable to subsequent injury [28]. In addition, MET could inhibit glutamate-induced excitotoxicity in neurons and attenuate stroke-induced nitrative signaling in diabetic rats [29,30]. Through these multiple mechanisms, MET may provide robust protection against ischemic injury and, thereby, improve stroke outcomes in DM patients receiving EVT. However, caution should be exercised in generalizing our results because our study and others have shown that neither metformin nor lifestyle interventions reduced major cardiovascular events over 20 years despite long-term diabetes prevention [31]. Future studies should take into account the multiple and complex mechanisms of MET in stroke [11]. Although we could not establish causality in our study, it is possible that the association between MET and initial infarct volume in this study may explain some of the observed associations. Further elucidation of the molecular mechanisms governing MET-mediated neuroprotection may reveal novel therapeutic targets for the treatment of ischemic stroke and other neurodegenerative diseases.

Interestingly, we showed that the MET dose could not show a dose–response effect on END-prog, END-SHT and the 3-month mRS of 0 to 2 in DM patients receiving EVT. Consistent with our finding, a previous study showed that MET dose was not associated with initial NIHSS, 3-month mRS, SHT and mortality in patients receiving IVT [16]. There was little clinical evidence to evaluate stroke outcomes according to the MET dose. An experimental study showed that a high dose of MET resulted in reduced reperfusion injury, smaller infarct size and improved left ventricular geometry, accompanied by the upregulation of AMPK and endothelial nitric oxide synthase expression [26,31]. In contrast, some conflicting evidence has been reported from experimental studies. The administration of a high dose of MET did not reduce mortality in a mouse model of myocardial infarction [32,33]. In addition, MET did not reduce infarct size and left ventricular ejection fraction in the porcine model [34]. The high dose of MET may induce prolonged astrocytic glycolysis and subsequent progressive lactic acidosis [28]. These discrepancies indicate the complex interplay between MET dosage, pathological processes, and the heterogeneity of experimental models. Therefore, the effects of MET dosage on stroke outcomes in our study should be generalized with caution. Future studies should explore the mechanistic basis of dose-dependent MET effects, investigate potential interactions with other therapeutic agents, and evaluate long-term clinical outcomes in larger prospective cohorts.

Although this is the first study to evaluate the effect of MET on stroke prognosis in patients receiving EVT, some limitations should be discussed. First, we could not confirm the causal relationship between MET and stroke outcomes because of the observational nature of the study. Second, we could not exclude unmeasured confounders and could not perform propensity score matching to adjust for variables because of the relatively small sample size. However, demographic variables and vascular risk factors were generally not different between the two groups, and we adjusted for these variables in the analysis. Third, our study did not evaluate the duration of MET therapy, which may influence its neuroprotective effects.

In conclusion, this study first highlights the possible prophylactic protective effects of prestroke MET use in patients with acute ischemic stroke and DM, resulting in a lower rate of END and better functional outcomes after EVT. These findings support the further investigation of the potential role of MET as an adjunctive therapy in this high-risk population. Future research should focus on elucidating the underlying mechanisms of the neuroprotective effects of MET and evaluating its long-term impact on functional outcomes and quality of life in stroke survivors.

## Figures and Tables

**Table 1:** Baseline characteristics between MET+ and MET− groups.

	MET− (*n* = 94)	MET+ (*n* = 137)	*p*-Value
Age, year (SD)	72.5 (13.1)	71.2 (11.5)	0.46 ^†^
Male, *n*	49 (52.1)	76 (55.5)	0.69 *
Initial NIHSS score (IQR)	15 (9)	13 (9)	0.46 ^‡^
Time interval from onset to arrival, hour (IQR)	2.3 (5.3)	1.8 (3.6)	0.44 ^‡^
Time interval from arrival to puncture, min (IQR)	121 (76)	123 (66)	0.71 ^‡^
Stroke subtypes, *n*			0.09 *
LAA	20 (21.3)	41 (29.9)	
CE	55 (58.5)	81 (59.1)	
others	19 (20.2)	15 (10.9)	
ASPECT score, (IQR)	8 (2)	8 (2)	0.51 ^‡^
Prior stroke, *n* (%)	32 (34.0)	34 (24.8)	0.14 *
Hypertension, *n*	68 (72.3)	94 (68.6)	0.56 *
Hyperlipidemia, *n*	26 (27.7)	32 (23.4)	0.54 *
Current smoking, *n*	13 (13.8)	18 (13.1)	1.00 *
Atrial fibrillation, *n*	50 (53.2)	73 (53.3)	1.00 *
Prior antiplatelet use, *n*	25 (26.6)	37 (27.0)	1.00 *
Prior anticoagulation, *n*	19 (20.2)	28 (20.4)	1.00 *
Collateral status, *n*			0.44 *
Good	52 (55.3)	84 (61.3)	
Intermediate	37 (39.4)	43 (31.4)	
poor	5 (5.3)	10 (7.3)	
Successful reperfusion, *n*	81 (86.2)	116 (84.7)	0.85 *
Occlusion site, *n*			0.85 *
M1	81 (86.2)	115 (83.9)	
M2	8 (8.5)	15 (10.9)	
ICA	5 (5.3)	7 (5.1)	
Infarct volume, mL (IQR)	30.5 (104)	13 (57)	0.04 ^‡^
HbA1c, % (SD)	7.2 (1.6)	7.5 (1.4)	0.37 ^†^
Creatinine, mg/dL (SD)	1.2 (1.2)	1.1 (0.6)	0.11 ^†^
Hemoglobin, mg/dL (SD)	13.6 (2.1)	13.5 (2.0)	0.63 ^†^
LDL, mg/dL (SD)	47.9 (31.4)	48.4 (31.8)	0.76 ^†^
Prothrombin time, INR (SD)	1.03 (0.18)	1.06 (0.29)	0.11 ^†^
Initial random glucose, mg/dL (SD)	175.6 (86.7)	180.8 (70.1)	0.26 ^†^
Systolic blood pressure, mmHg (SD)	151.9 (28.3)	151.7 (26.5)	0.58 ^†^

Abbreviation: MET−, no prestroke metformin use; MET+ prestroke metformin use; SD, standard deviation; NIHSS, National Institute Health Stroke scale; IQR, interquartile range; ASPECT, Alberta Stroke Program Early CT Score; LAA, large artery atherosclerosis; CE, cardioembolism; M1/M2, middle cerebral artery; ICA, internal carotid artery; HbA1c, glycated hemoglobin; LDL, low-density lipoprotein cholesterol. * Calculated using the chi-square test, ^†^ Calculated using Student’s *t*-test, ^‡^ Calculated using Mann–Whitney U test.

**Table 2:** Multivariate analysis showing impact of MET+ on stroke outcomes after EVT.

	END-Prog			END-SHT			3-Month mRS Score of 0 to 2		
	OR	95% CI	*p*-Value	OR	95% CI	*p*-Value	OR	95% CI	*p*-Value
MET+	0.24	0.12–0.48	<0.001	0.33	0.14–0.75	0.01	2.80	1.27–6.21	0.01
Age	1.01	0.98–1.04	0.45	1.06	1.01–1.11	0.02	0.97	0.94–0.999	0.04
Male	1.07	0.54–2.12	0.84	1.50	0.63–3.54	0.36	1.33	0.64–2.75	0.44
Initial NIHSS	0.99	0.94–1.05	0.83	1.03	0.96–1.10	0.43	0.94	0.88–0.99	0.03
Time interval from onset to arrival	0.95	0.88–1.02	0.14	1.02	0.96–1.09	0.50	0.89	0.79–0.996	0.04
Time interval from arrival to puncture	1.001	1.00–1.003	0.13	1.001	0.999–1.003	0.41	0.997	0.99–1.00	0.054
Stroke subtypes									
Other	reference			reference			reference		
LAA	1.55	0.54–4.46	0.42	0.44	0.10–1.90	0.27	1.07	0.37–3.10	0.91
CE	1.68	0.64–4.40	0.29	0.81	0.26–2.55	0.72	0.94	0.35–2.53	0.90
Prior stroke	0.53	0.25–1.13	0.10	0.53	0.21–1.33	0.17	2.86	1.31–6.26	0.01
glycated hemoglobin	1.17	0.92–1.49	0.21	0.99	0.72–1.35	0.93	0.92	0.71–1.20	0.55
Creatinine	0.89	0.61–1.28	0.52	1.54	1.03–2.32	0.04	0.39	0.15–0.99	0.046
Prothrombin time	0.31	0.04–2.28	0.25	0.88	0.12–6.36	0.90	1.30	0.38–4.46	0.67
Initial random glucose	0.998	0.99–1.002	0.35	1.001	0.995–1.01	0.75	0.995	0.99–1.001	0.12
Collateral status	1.43	0.86–2.38	0.17	1.56	0.84–2.89	0.16	0.86	0.49–1.52	0.61

Abbreviation: MET+ prestroke metformin use; EVT, endovascular treatment; OR, odd ratio; CI, confidence interval; END-prog, stroke progression; END-SHT, symptomatic hemorrhagic transformation; mRS, modified Rankin Scale; NIHSS, National Institute of Health Stroke Scale; LAA, large artery atherosclerosis; CE, cardioembolism.

**Table 3:** Multivariate analysis showing the effect of MET dose on stroke outcomes after EVT.

	END-Prog			END-SHT			3-Month mRS Score of 0 to 2		
	OR	95% CI	*p*-Value	OR	95% CI	*p*-Value	OR	95% CI	*p*-Value
MET dosage									
No MET	reference			reference			reference		
<1000 mg daily	0.06	0.02–0.23	<0.001	0.27	0.09–0.84	0.02	1.74	0.65–4.66	0.27
1000~2000 mg daily	0.35	0.15–0.80	0.01	0.41	0.131.24	0.11	4.17	1.67–10.40	0.002
>2000 mg daily	0.51	0.18–1.41	0.19	0.31	0.06–1.63	0.17	2.70	0.81–8.92	0.11

Abbreviation: MET, metformin; EVT, endovascular treatment; END-prog, stroke progression; END-SHT, symptomatic hemorrhagic transformation; mRS, modified Rankin Scale; OR, odd ratio; CI, confidence interval.

## Data Availability

The data that support the findings of this study are available upon reasonable request from the corresponding author.

## Supplementary Materials

The following supporting information can be downloaded at: https://www.mdpi.com/article/10.3390/biomedicines12040745/s1, Figure S1: Distribution of HbA1c values on admission in percent presented as medians with interquartile ranges according to MET dose, Table S1: Multivariate analysis showing effect of MET dose on stroke outcomes after EVT (including adjusted covariates), Table S2: Multivariate analysis showing effect of MET monotherapy on stroke outcomes after EVT.
