# Supplementary material for: Effects of Prior Metformin Use on Stroke Outcomes in Diabetes Patients with Acute Ischemic Stroke Receiving Endovascular Treatment
Source: Biomedicines. 2024 Mar 27;12(4):745. doi: 10.3390/biomedicines12040745 (PMC11048027; doi:10.3390/biomedicines12040745)
Supplement: Supplementary file 1 [file biomedicines-12-00745-s001.zip › biomedicines-2914923-supplementary.pdf]

Supplementary Figure S1. Distribution of HbA1c values on admission in percent presented as medians with interquartile ranges according to MET dose.

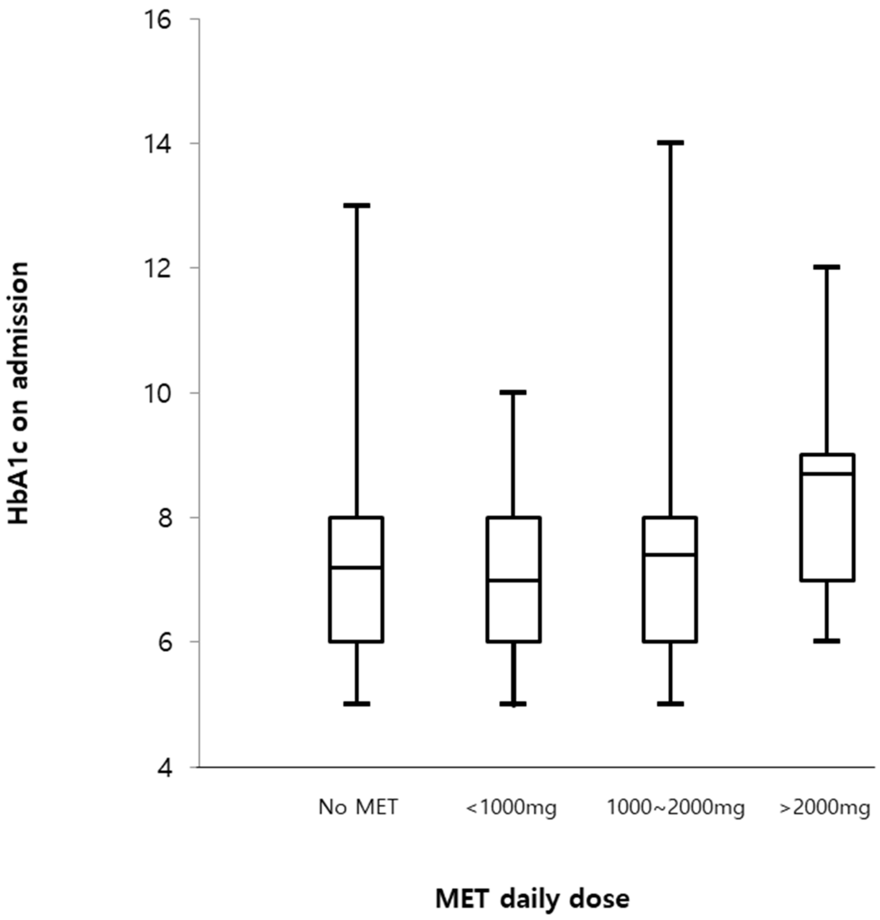

Supplementary Table S1. Multivariate analysis showing effect of MET dose on stroke outcomes after EVT (including adjusted covariates).

	END-prog		END-SHT		3-month mRS 0-2	
	OR	95%CI	OR	95%CI	OR	95%CI
MET dose						
No MET	reference		reference		reference	
<1000mg daily	0.06	0.02-0.23	0.27	0.09-0.84	1.74	0.65-4.66
1000~2000mg daily	0.35	0.15-0.80	0.41	0.13-1.24	4.17	1.67-10.40
>2000mg daily	0.51	0.18-1.41	0.31	0.06-1.63	2.70	0.81-8.92
Age	1.01	0.98-1.05	1.06	1.01-1.11	0.97	0.94-1.00
Male	1.09	0.54-2.17	1.50	0.63-3.53	1.46	0.70-3.08
Initial NIHSS	0.99	0.94-1.05	1.03	0.96-1.10	0.93	0.88-0.99
Time interval from onset to arrival	0.94	0.87-1.02	1.02	0.96-1.09	0.88	0.79-0.995
Time interval from arrival to puncture	1.002	1.00-1.003	1.00	0.999-1.003	0.997	0.99-1.00
Stroke subtypes						
Other	reference		reference		reference	
LAA	1.46	0.49-4.28	0.43	0.10-1.89	1.11	0.38-3.25
CE	1.60	0.61-4.23	0.82	0.26-2.58	0.99	0.36-2.69
Prior stroke	0.53	0.25-1.12	0.52	0.21-1.31	2.86	1.30-6.33
HbA1c	1.10	0.85-1.42	0.99	0.72-1.37	0.92	0.69-1.23
Creatinine	0.93	0.66-1.33	1.57	1..02-2.42	0.39	0.15-1.05

Prothrombin time	0.32	0.05-2.31	0.91	0.13-6.59	1.32	0.37-4.72
Initial random glucose	0.998	0.99-1.00	1.00	0.995-1.01	0.995	0.99-1.00
Collateral status	1.43	0.85-2.42	1.53	0.82-2.86	0.79	0.44-1.43

Supplementary Table S2. Multivariate analysis showing effect of MET monotherapy on stroke outcomes after EVT.

	END-prog		END-SHT		3-month mRS 0-2	
	OR	95% CI	OR	95% CI	OR	95% CI
MET monotherapy	0.20	0.09-0.43	0.25	0.10-0.64	2.92	1.25-6.86
Age	1.01	0.98-1.04	1.05	0.99-1.10	0.98	0.94-1.01
Male	1.32	0.63-2.76	1.51	0.61-3.76	1.15	0.53-2.51
Initial NIHSS	0.998	0.94-1.06	1.01	0.91-1.08	0.93	0.88-0.996
Time interval from onset to arrival	0.94	0.87-1.01	1.03	0.97-1.10	0.88	0.78-0.995
Time interval from arrival to puncture	1.001	1.00-1.003	1.001	0.999-1.003	0.997	0.995-1.00
Stroke subtypes						
Other	reference		reference		reference	
LAA	2.03	0.65-6.32	0.36	0.07-1.75	0.99	0.32-3.09
CE	1.93	0.69-5.41	0.83	0.26-2.66	0.85	0.30-2.42
Prior stroke	0.46	0.20-1.03	0.54	0.20-1.45	3.13	1.35-7.28
HbA1c	1.20	0.93-1.55	1.03	0.75-1.42	0.87	0.65-1.15
Creatinine	0.90	0.63-1.29	1.50	1.01-2.23	0.36	0.13-0.99
Prothrombin time	0.41	0.05-3.54	1.43	0.14-14.19	1.89	0.40-9.01
Initial random glucose	0.997	0.99-1.002	1.001	0.995-1.01	0.995	0.99-1.002

Collateral status	1.63	0.93-2.87	1.36	0.70-2.67	0.75	0.38-1.46
